# Editorial

**DOI:** 10.1093/braincomms/fcaa225

**Published:** 2021-01-16

**Authors:** Tara Spires-Jones

**Affiliations:** Edinburgh, UK

Welcome to Volume 3, issue 1 of *Brain Communications*. While 2020 has been a tumultuous and difficult year, our journal has luckily continued to flourish. We are proud of the breadth of rigorous translational neuroscience studies that are being submitted to our journal. We have continued to publish papers on a wide range of conditions from neurodevelopmental disorders to neurodegeneration and studies ranging from fundamental neurobiology to diagnostic tools and results of clinical trials (see e.g. Landles *et al.*, 2020; Luz *et al.*, 2020; Maekawa *et al.*, 2020; Ugalde *et al.*, 2020; Van Damme *et al.*, 2020). We have also published a few timely papers on the neurological and cognitive effects of COVID-19 (Lleó and Alcolea, 2020; Ritchie *et al.*, 2020; Woo *et al.*, 2020). Submissions in the first three quarters of 2020 were on average around 58 papers per month, up from 35 per month in the last half of 2019. Some of these are likely due to our growing reputation, and some are likely due to the lockdown-induced data analysis and write up mania while many of us around the world were locked out of our labs during the pandemic. In addition to primary research papers and reviews, we have published several scientific commentaries and letters to the editor about our papers, and I look forward to continuing this discourse between readers, authors, and the wider field about the implications and interpretations of data published in *Brain Communications*.

As further evidence of the quality of our authors’ work and the efforts of our publisher Oxford University Press, our application for inclusion in PubMed Central (PMC) was successful, meaning that all of our papers now automatically appear in PMC and are searchable in PubMed. We are also now indexed in the Emerging Sources Citation Index and the Directory of Open Access Journals, which are important steps for a new open access journal to build a reputation in the scholarly publishing space.

Our wonderful team of editors have been working hard to keep your papers moving without too much delay despite the difficulties finding reviewers with time to help review papers during the pandemic. Our average time to first decision on papers is around 30 days. Over the past 6 month, we have said goodbye to several editors who have stepped down due to retirement or other commitments, and I extend heartfelt thanks to all of them for helping to launch the journal. Thank you Richard Ribchester, Asya Rolls and Kevin Talbot! We are thrilled to welcome several new editorial board members to the team (Alfred Njamnshi, Paul Skehel, Graciela Muniz Terrera, David Sharp and Anne Rosser). For a list of all of our team members and information about their areas of expertise, please see https://academic.oup.com/braincomms/pages/Editorial_Board (last accessed 16 December 2020).

There are also big changes happening this year with our sister Journal *Brain* as former Editor in Chief Dimitri Kullmann has finished his term and handed over the reins to Masud Husain. Masud and his new editorial team have refreshed *Brain’s* style including changing the format of references in papers. To make transfers from *Brain* to *Brain Communications* as seamless as possible, we will be changing our referencing style to match.

The cover image for this issue comes from the review by Jonathan Lifshitz and colleagues, which reminds us of the importance and beauty of rod morphology adopted by some microglia after injury, which had been largely ignored for almost 100 years (Giordano *et al.*, 2021). In this review, scientific illustrator Phoebe Dubisch sketched rod microglia that appear in rat brain after diffuse traumatic brain injury, highlighting the elongated, twisted soma and processes.

Thank you all for continuing to support our journal by sending in papers, reading the work here and spreading the word. Wishing you all a healthy 2021 full of vaccines and eventually, in-person conferences and even hugs!
